# The Week's Parliament and Publications

**Published:** 1910-07-02

**Authors:** 


### The Week's Parliament and Publications.

### MEDICAL OFFICERS AND THE TOWN PLANNING ACT.

Mr. John Burns has announced that the general order prescribing the duties of medical officers of health under the Housing, Town Planning, &c., Act, 1909, is in an advanced state of preparation, and will be issued shortly.

### THE BUDGET.

Medical practitioners have been eagerly wondering whether the Budget will contain a decrease in the spirit duty. We leave prophecies to the lay newspapers; but it is necessary to remind our readers that very persistent rumours are current, rumours which deny the decrease desired. In any case the question will be settled and the truth known on Thursday night.

### ALLEGED DANGEROUS TRADES.

Since the publication of the report of the Chief Inspector of Factories and Workshops, referred to below, the Home Office has informed the Secretary of the Trade Union Congress that Dr. Legge has been unable to find that there is any ground for the assumption that either copper or brass are poisonous substances within the meaning of the Factory Act 1901. He has also been unable to find that chlorinous fumes in dyeworks are poisonous.

### THE BACILLUS OF ANTHRAX.

It appears from a reply given by Sir Edward Strachey, in answer to a question put by Mr. Bathurst, that the Board of Agriculture have reason to believe that a considerable proportion of the cases now returned as anthrax are not so in fact; but as in all cases where the cause of death is diagnosed as anthrax the anima, s carcase is immediately destroyed, it is seldom possible to inquire fully into the cause of death. The bacillus of anthrax is easy to identify when present in fresh blood, but it is extremely difficult to exclude its presence when the animal has been dead for several hours.

### CORONERS' LAW AND DEATH CERTIFICATION.

The Bill to amend the law relating to coroners' law and the certification and registration of deaths and burial, which has been introduced in the House of Commons by Sir William Collins, is now in the hands of members. Its intention is to remove certain anomalies disclosed by reports of several committees during recent years. Thus in 1893 a Select Committee was appointed to "inquire into the sufficiency of the existing law as to the disposal of the dead, for securing an accurate record of the causes of death in all cases, and especially for detecting whether death may have been due to poisoning, violence, or criminal neglect." The report of that Committee indicated the urgent necessity for reform. The Inter-Departmental Committee on Physical Deterioration, which sat in 1903, also directed attention, in their report, to the dangers incidental to the defects in the law relative to the registration and certification of deaths, and recommended the registration of still-births. In 1908 a Departmental Committee of the Home Office was appointed to inquire into the law relating to coroners and coroners' inquests, and into the practice in coroners' courts, and their report was published some weeks ago (Cd. 5004). The provisions in Sir William Collins' Bill are intended to give effect to many of the recommendations of the Departmental Committee of 1908 and Deaths Certification Committee of 1893. Part I. of the Bill, which consists of fifteen clauses, deals with the appointment, payment, retirement, superannuation, and qualification of coroners. It also provides that a coroner after due inquiry into any case referred to him may decide not to hold an inquest if he is satisfied that the deceased died a natural death, and provides that it shall not be obligatory for the jury at any inquest to view the body unless the coroner shall deem it necessary. County and borough councils are empowered to appoint one or more medical investigators or pathologists in each coroner's district to assist the coroner in his inquiries and inquests, and to make post-mortem examinations. Such investigators to be paid out of the county or borough rate. Part II., which consists of ten clauses, makes provision for the registration and certification of death and burial. It provides that no death shall be registered under the Registration Acts without the delivery to the registrar of a certificate of death duly signed by a registered practitioner or by a coroner after holding an inquiry. It requires that, before giving a certificate, a registered medical practitioner shall personally inspect the body and identify it as the body of the person named in the certificate, and shall certify to the fact of death as well as to its cause. Every practitioner who gives a death certificate is required within twenty-four hours after the death takes place to send the certificate to the local registrar of deaths.

### EXPERIMENTS ON LIVING ANIMALS.

A report has been issued showing the number of experiments on living animals during the year 1909. The total number of persons who held licences in that year was 483, of whom 135 performed no experiments. The report furnishes evidence that licences and certificates have been granted and allowed only upon the recommendation of persons of high scientific standing; that the licensees are persons who, by their training and education, are fitted to undertake experimental work and profit by it; that all experimental work has been conducted in suitable places. The total number of experiments performed was 86,277, being 2,357 less than in 1908. The vast majority of the experiments—viz. 82,389—were performed without anæsthetics, these being mostly inoculations, but a few were feeding experiments or the administration of various substances by the mouth or by inhalation, or the abstraction of blood by puncture or simple venesection. In no instance has a certificate dispensing with the use of anæsthetics been allowed for an experiment involving a serious operation. 2,184 experiments came under the regulation which provides that the animal must be kept under an anæsthetic during the whole of the experiment, and must, if the pain is likely to continue after the effect of the anæsthetic has ceased, or if any serious injury has been inflicted on the animal, be killed before it recovers from the influence of the anæsthetic. During the year 45,182 experiments, mainly inoculations, into mice, were performed at five institutions in the course of cancer investigations.

### INDUSTRIAL POISONING.

The annual report of the Chief Inspector of Factories and Workshops for the year 1909 contains much that is of medical interest. It would not be possible in a brief summary to do more than refer to a few of the main points. It is satisfactory to note that as regards cleanliness there has been a fair amount of improvement in the way in which factories and workshops are kept. The general use of limewash for the walls and ceilings, in preference to paint or varnish, is noted, but neglect to apply it within the statutory period is a frequent source of complaint. Dealing at length with the notification of lead-poisoning during the past ten years, Dr. Legge (Medical Inspector) points out that, taking the total figures, there has been a reduction of more than 50 per cent. in the number reported. The principal decrease is limited to those trades or processes in which exhaust ventilation for removal of dust has been applied, *i.e.* white lead, earthenware and china, litho-transfers, and paint and colours. On the other hand, in smelting and in industries using paint, where hitherto it has been found difficult or impossible to apply local exhaust, there has been some increase. There is also a perceptible increase in coach-painting which is probably due to some extent to the motor-car industry. Notwithstanding the substantial decrease in the number of cases of lead-poisoning reported, it is a remarkable fact that the number of fatal cases ascribed to lead-poisoning has not decreased. As to mercurial poisoning, symptoms were found in workers engaged in drying and mixing fulminate of mercury in a cartridge-filling factory, and in the manufacture of electric mercury meters. Another case was reported from a card-clothing factory. The teeth of the cards are tempered by passing them through a mercury bath; some of the mercury adheres and is given off as dust or by evaporation in the subsequent processes. There was an increase in the number of cases of anthrax, and the mortality is twice as high among those who contracted the disease from hides as from wool, though the number of cases is smaller. Workers employed in the manufacture of roll tobacco have been found to be troubled with eczema on the hands. Careful inquiry in several factories warranted the conclusion that the oil used in the process was the cause. Dr. Legge thinks that care in the methods of adding the oil to the tobacco will diminish the injurious effects. Cases of arsenic poisoning numbered four, as compared with 23 in 1908. Careful analysis was made in the well-equipped laboratory of a large chemical works to ascertain if any appreciable amount of arsenic was present in the urine of men exposed to the possibility of arsenic absorption, but who appeared to be in perfect health. The urine of four such persons showed amounts varying from 0.0011 to 0.0035 grain per gallon. Such amounts, in the opinion of Dr. Legge, could not conceivably affect health.

